# Nerve degeneration and regeneration in the cephalopod mollusc *Octopus vulgaris*: the case of the pallial nerve

**DOI:** 10.1038/srep46564

**Published:** 2017-04-20

**Authors:** Pamela Imperadore, Sameer B. Shah, Helen P. Makarenkova, Graziano Fiorito

**Affiliations:** 1 Department of Biology and Evolution of Marine Organisms, Stazione Zoologica Anton Dohrn, 80121 Napoli, Italy; 2 Association for Cephalopod Research – CephRes, 80133 Napoli, Italy; 3 Departments of Orthopaedic Surgery and Bioengineering, University of California, San Diego, La Jolla, CA 92093, USA; 4 Department of Cell and Molecular Biology, Scripps Research Institute, San Diego, La Jolla, CA 92037, USA

## Abstract

Regeneration is a process that restores structure and function of tissues damaged by injury or disease. In mammals complete regeneration is often unsuccessful, while most of the low phyla animals can re-grow many parts of their body after amputation. Cephalopod molluscs, and in particular *Octopus vulgaris*, are well known for their capacity to regenerate their arms and other body parts, including central and peripheral nervous system. To better understand the mechanism of recovery following nerve injury in this species we investigated the process of axon regrowth and nerve regeneration after complete transection of the *Octopus* pallial nerves. This injury induces scar formation and activates the proliferation of hemocytes which invade the lesion site. Hemocytes appear involved in debris removal and seem to produce factors that foster axon re-growth. Connective tissue is involved in driving regenerating fibers in a single direction, outlining for them a well-defined pathway. Injured axons are able to quickly re-grow thus to restoring structure and function.

Cephalopod molluscs are known for the richness and plasticity of their behavioral repertoire and underlying neural control^1^,^2^,^3^,^4^. These animals possess the remarkable ability to heal and regenerate a variety of different tissues^5^,^6^,^7^. Recent work on cuttlefish^8^,^9^, squid^10^,^11^ and octopus^12^,^13^ resumed interest towards this aspect of cephalopods biology, providing new information available. Despite recent experimental studies, detailed knowledge about cellular and molecular mechanisms occurring during the different phases of regeneration in cephalopods is still missing.

The common octopus, *Octopus vulgaris,* is known to heal and regenerate appendices, cornea, peripheral nerves and neural pathways within the central nervous systems^5^,^7^,^14^,^15^,^16^,^17^. After their original observations Sereni^18^ and Young^19^ were the first to provide a description of the histological and physiological phenomena occurring in octopus after severing one of the two mantle connectives, i.e. the pallial nerve^17^, thus to lesion one side only. Although several cephalopod species were studied in this work, most of the accounts were based upon observations carried out on octopuses (*O. vulgaris* and *Eledone moschata*). A functional recovery was observed only in six cases over 200 lesioned animals, with the earliest signs of functional regeneration seen 65 days post operation^17^. In a series of successive works a better description of the morphological and physiological events occurring after sectioning one of the pair of octopus’ pallial nerves was provided^14^,^20^. Subsequent studies provided additional insights^21^,^22^,^23^. In particular, Authors described a series of morphological (i.e. amoebocytes contributing to scar formation and infiltrating the stumps; axon degeneration and regeneration of fibers) and physiological events (i.e. amitotic proliferation, phagocytosis) as summarized in Table 1.

*O. vulgaris* possesses a pair of pallial nerves, one for each side of the mantle; these originate from the palliovisceral lobe located at the posterior end of the subesophageal mass, a part of the central nervous system^15^,^24^. Each of the nerves runs inside a “muscular bridge”, as originally described by Sereni^17^, facing the internal cavity of the mantle and ends in the stellate ganglion. Each ganglion gives rise to stellar nerves, which in turn innervate the mantle (Fig. 1a,b).

Each nerve fiber in the pallial nerve is enveloped by connective tissue sheaths; an additional outer connective layer enwraps the whole nerve^17^,^20^,^25^. Here we do not provide a throughout description of ***i.*** the organization of fibers in the pallial nerve^15^,^24^, and ***ii.***the structure and organization of the stellate ganglion^20^,^26^,^27^ of *O. vulgaris*.

Through the innervation of muscles and chromatophores, each pallial nerve provides neural network control for breathing and skin patterning on the ipsilateral side of the mantle.

The transection of a nerve leads to immediate paralysis of respiratory mantle muscle on the denervated side, chromatophore relaxation and consequent paling of the mantle^14^,^17^,^28^. The complete regeneration of the pallial nerve and functional recovery require approximately three to four months^14^.

Here we describe the sequence of events occurring after complete transection of *O. vulgaris* pallial nerve within the first 14 days after lesion. We limited our study to the first two weeks since our preliminary data revealed this to be sufficient for the reorganization of the damaged structures leading the two stumps to occur. This time frame also led to a functional recovery observed at the level of behavioral outcomes after the lesion.

Degeneration and regeneration are evaluated through morphological and functional analyses in the same individual by comparing the two pallial nerves: one post-lesion, and the contralateral one exposed and left intact, thus serving as sham control. We also describe the cellular events linked to nerve regeneration, the existence of inflammatory events, cell proliferation and the first signs of functional recovery.

## Results

All animals behaved normally and did not exhibit any sign of distress or suffering after surgery^29^,^30^,^31^ and during the following 14 days post-lesion (p.l.). Octopuses attacked their prey promptly in less than 30 seconds in all occasions exhibiting their normal predatory behavior^32^,^33^,^34^ and fed regularly.

### Behavioural patterns

A full attack response^3^,^35^ was observed as response to the presentation of a crab as natural prey^32^,^34^. The effects of the damage due to the lesion appeared mostly on the mantle, ipsilateral to the lesioned side, as deficits in the full expression of body patterns exhibited by the animal.

**Figure 1:**
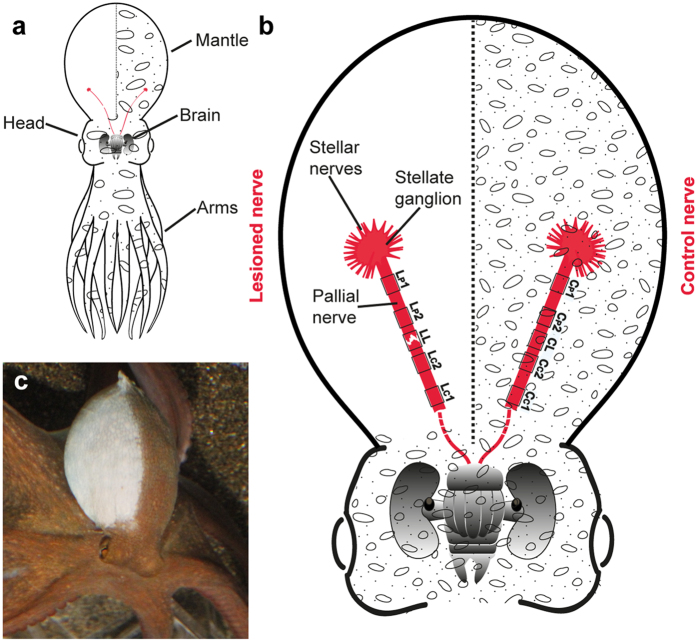
Main features of the anatomy of *Octopus vulgaris* in relation to the pallial nerve, and effects of its lesion. (**a**) General anatomy of *O. vulgaris*. In red the two pallial nerves are visible rising from the posterior part of the brain, each of them directed toward the ipsilateral side of the mantle. (**b**) Enlargement of head and mantle to show the two pallial nerves ending each into a stellate ganglion. For each nerve five areas are identified for the purpose of this study, and used as target for imaging and subsequent analysis: a distal and a proximal area in the central stump (respectively identified as L_C_1 and L_C_2), similarly in the peripheral stump (L_P_1 and L_P_2) of the transected nerve. Analogous areas were identified in the control nerve, i.e.: the one corresponding to the lesion site (CL), a distal and a proximal area in the central ‘stump’ (C_C_1, C_C_2), and at the level of the peripheral ‘stump’ (C_P_1, and C_P_2). (**c**) Right pallial nerve transection causes complete paling of the mantle at the level of the denervated side due to loss of neural control of the chromatic and textural components of body patterns. Body parts are easily recognizable (see **a**,**b**).

**Figure 2:**
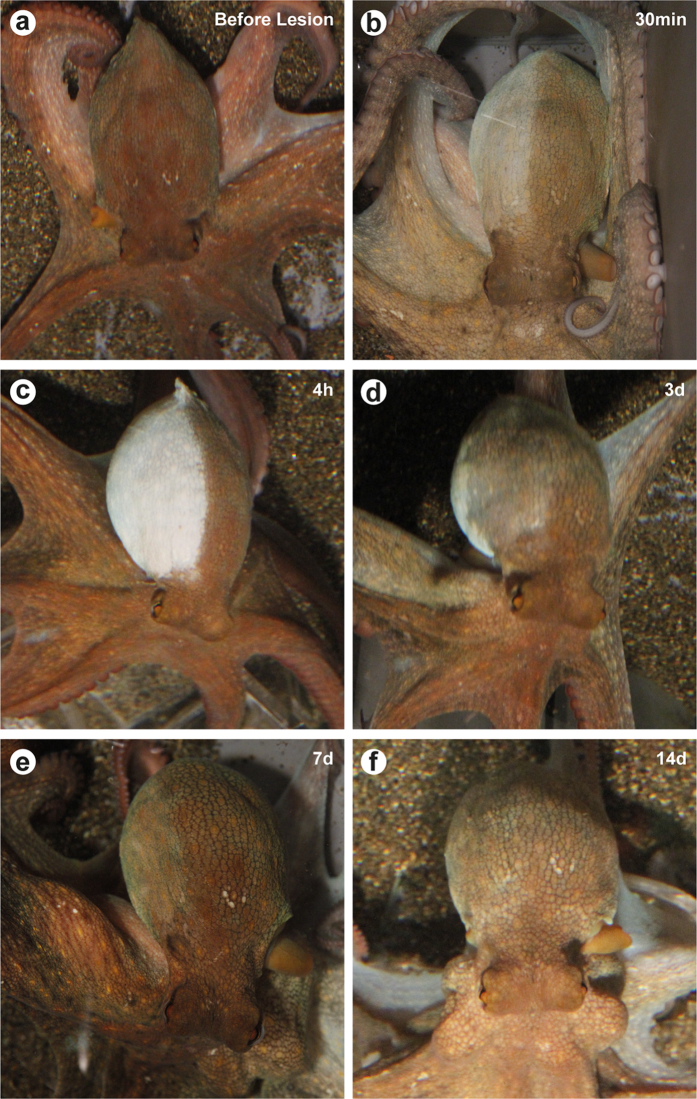
Behavioral patterns exhibited by octopuses following injury. (**a**) Before lesion an octopus showing normal body patterning. (**b**,**c**) Immediately after nerve transection the skin of the mantle of the lesioned side becomes pale (**b**; 30 min p.l.) and white (**c**; 4 h p.l.). Skin of the contralateral side retains the ability to perform full range of patterns as before lesion. (**d**) Three days post lesion uniform coloration is visible on dorsal area of the denerved area. (**e**,**f**) Octopuses at rest exhibit a colour pattern matching the contralateral side appearing improved with time after 7d (**e**) and 14d (**f**) after lesion.

After surgery, the mantle skin at the lesioned side appeared ‘pale’^3^,^28^ and no control of chromatic and textural pattern resulted evident (from 30 min to 24 h p.l.; Fig. 1c; Fig. 2c). The contralateral side retained the ability to perform the full range of body patterns as in a normal behaving animal^3^,^35^,^36^,^37^,^38^. A few hours after recovery from anesthesia some animals showed self-grooming actions^39^ consisting in arm bending over the head and the mantle surface^3^; grooming was exhibited in proximities of the denervated area and inside the mantle cavity.

One to two days later, light brown spots appeared on a white-greyish/pale background while three to five days post lesion the whole dorsal area of the denervated skin had a uniform colored appearance (Fig. 2d). Smooth skin texture was always observed in this phase.

In our experimental conditions, and contrary to what was previously described^14^, starting from seven days p.l. the animals exhibited at rest a marked ability of the mantle-skin to match the chromatic pattern of the uninjured side (Fig. 2e,f). Normal chromatic patterning was not observed on the mantle while the octopus performed an attack response, at all time-points (3, 7, 14d); in these occasions the lesioned side became again pale.

In addition, we observed immediately after lesion an impairment of the mantle contractions during normal breathing on the denervated side, with mantle muscle activity appearing jerky and unsynchronized. We did not notice improvement during the 14 days of this study in the capability of the mantle to contract on the lesioned side during breathing, despite the fact that muscular tone improved with time. We did not recognize any additional alterations in physiological outcomes due to the altered breathing motion, and no other evident bias in the motor patterns was detected in any octopus utilized in this experiment.

At sacrifice, anesthetized lesioned animals showed dark waves appearing apparently uncontrolled on a white-grayish background on the denervated area. These waves have been observed by Packard^40^ and referred as “wandering clouds”. They have been considered as due to hyper-excitability of the radial muscles of chromatophores^14^,^17^,^41^ and appear not to be under the control of the central nervous centers^18^. We rarely observed such waves in behaving animals. In addition, in the same conditions the denervated skin resulted to be very susceptible to mechanical stimuli contracting intensely, compared to the contralateral side which did not respond to stimulation.

### Hemocyte infiltration

**Figure 3:**
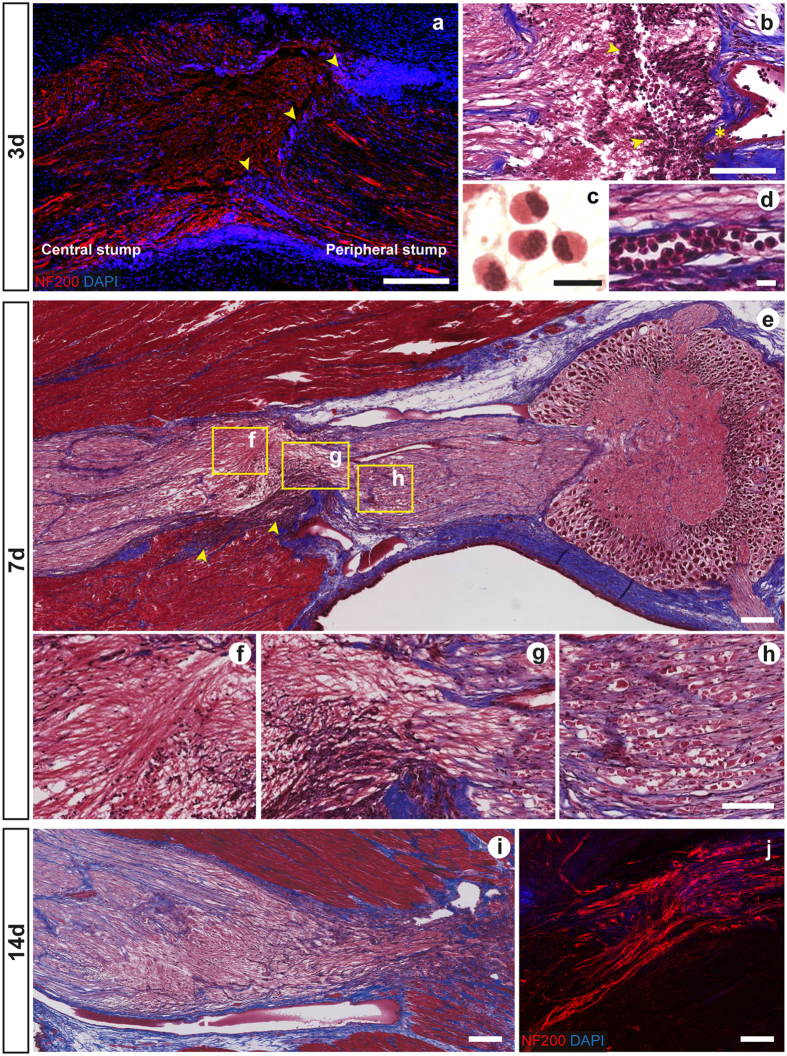
Degeneration and regeneration in the pallial nerve. (**a**) Following transection of the *O. vulgaris* right pallial nerve, a scar is visible 3d post-surgery separating the two stumps (marked with yellow arrows in **a**,**b**). Neurofilament (red) allows identification of nerve fibers, and DAPI (blue) marks cell nuclei. (**b**) Masson’s trichrome staining reveals scar tissue mainly formed by hemocytes which leave the blood stream and accumulate at the site of lesion (marked by asterisk). Regenerating fibers (pink), originating from the central stump, appear not enveloped by connective tissue and are seen to spread in several directions (**b**) toward the scar. (**c**) Hemocytes are identified in the hemorrhagic areas through hematoxylin and eosin staining (dark nuclei and pink cytoplasm). They were also found to infiltrate (**d**) in the connective tissue (in blue) and muscles (in red) lesioned to expose the nerve (after Masson’s trichrome staining). (**e**) Seven days (7d) post injury regenerating fibers from the central stump (**f**) overcome the scar and penetrate the opposite stump (**g**,**h**), the latter characterized by axon breakage. (**i**) Fibers of the central stump, 14 days post injury, regenerate forming a spike-like structure driven by connective tissue toward the opposite stump which, beside showing marked degeneration, presents bundles of regenerating axons (**j**). Scale bars: 200 μm in (**a**, **e–j**) (**b**): 100 μm; (**c** and **d)**: 10.

**Figure 4:**
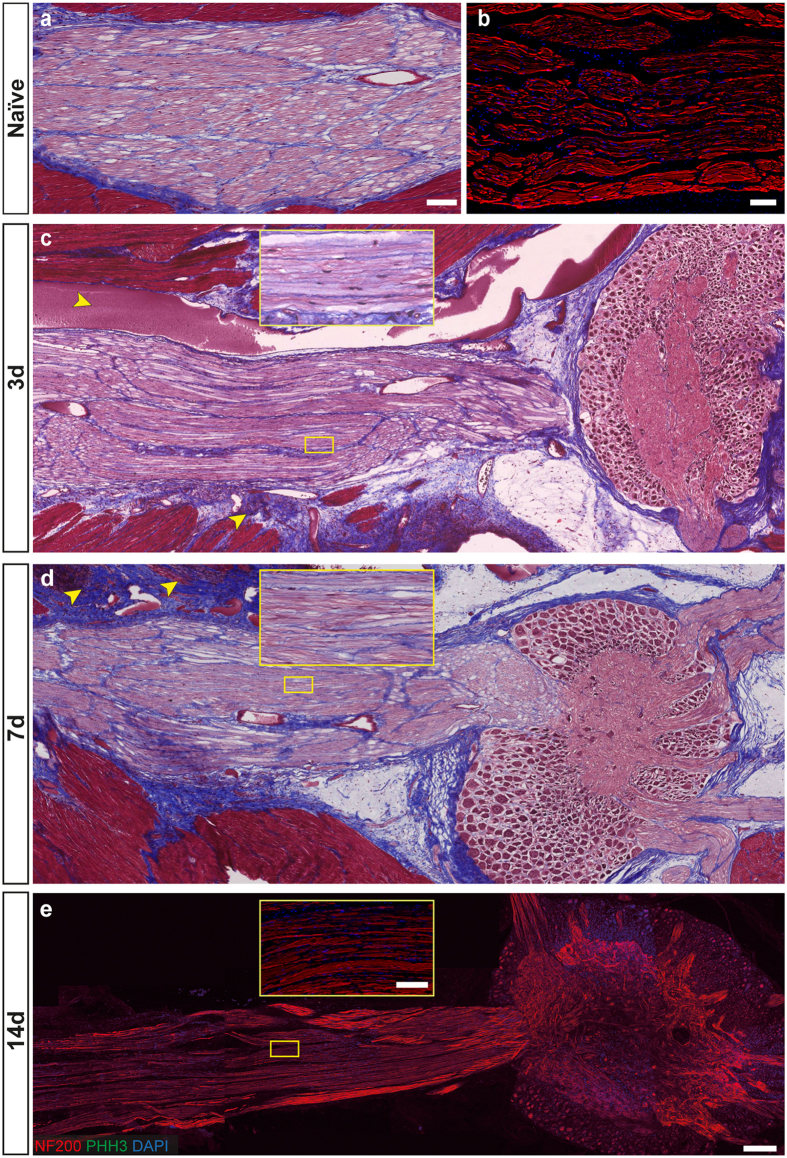
Naïve and sham control octopus pallial nerves. Control pallial nerve from naïve octopus (**a**,**b**) shows normal appearance of axons and connective tissue in an intact un-lesioned animal. Sham lesion of left pallial nerve in octopus does not alter nerve morphology; see enlargements in box for each nerve at the three time-points: (**c**) 3 days, (**d**) 7 days, and (**e**) 14 days post lesion. Hemorrhagic areas and scar tissue are identified in the lesioned connective tissue and muscles around the nerve. These are marked with yellow arrows in (**c** and **d)**. Only few mitotic cells were highlighted in sham control nerves, limited to the outer connective tissue layer (**c**). Scale bar: 100 μm in (**a**,**b);** 200 μm in (**c**–**e)**; yellow enlargements: 50 μm.

Large hemorrhagic areas were observed in the damaged connective tissue and muscle lesioned to expose the pallial nerves of both sides. This was probably due to the breakage of vessels that run along the pallial nerve^42^. Scar tissue appeared in all the injured areas, i.e. muscles, connective and nerve tissues, even though the extent of the cicatricial tissue in the sham control appeared minimal relative to the scars formed post lesion (Fig. 3 and 4).

Three days after surgery a thick scar appeared separating the cut ends of the nerve (Fig. 3a,b). Fourteen days post lesion a large reduction of the size of the scar was observed.

The scar impeded the two stumps to come into contact (Fig. 3a). However, this did not represent a permanent barrier for the axons outgrowth. Regenerating fibers from the central stump appeared growing into the scar toward the peripheral stump within seven days post lesion (Fig. 3f).

**Figure 5:**
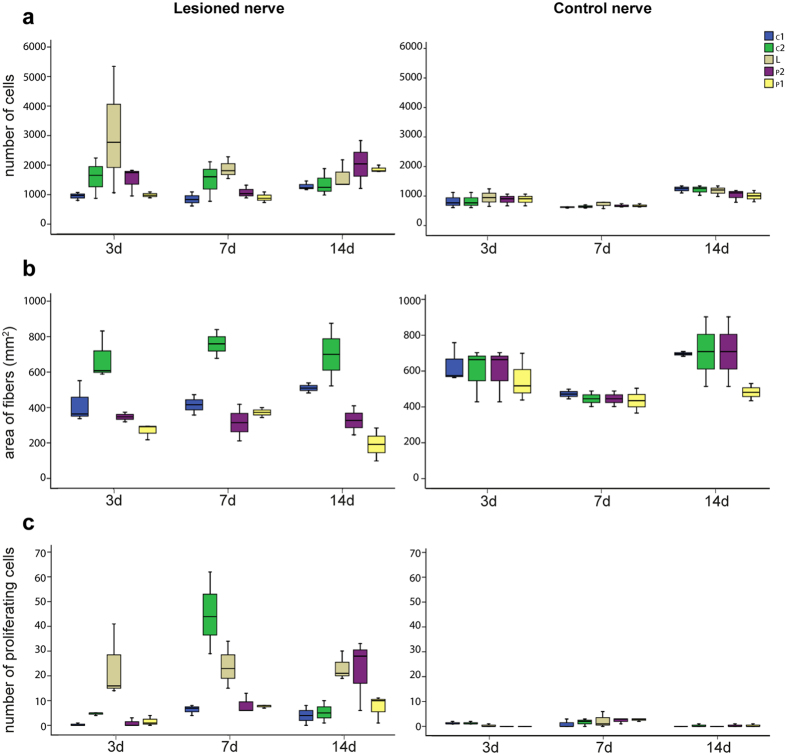
Boxplots showing (**a**) number of cells involved in scar formation, (**b**) nerve fibers degeneration/regeneration and (**c**) cell proliferation after octopus pallial nerve transection. Three days post lesion (3d), an increase in the number of cells (**a**) is observed in LL, consisting of scar formation and hemocytes infiltration (LL vs CL, p = 0.043 after Student’s *t*-test). The same area is also interested by intense mitotic activity. (**b**) L_C_2 area shows a high increase in nerve fiber area due to axon regeneration at all time-points investigated (at 7d, L_C_2 vs C_C_2 p < 0.010 after Student’s *t*-test), while degeneration is mainly observed in L_P_2 (at 3d, 7d and 14 days, L_P_2 vs C_P_2 p < 0.010 after Student’s *t*-test) and L_P_1 14d. (**c**) Cell infiltration and proliferation inside the cut nerve is also observed in L_C_2 seven (7d, L_C_2 vs C_C_2 p = 0.001 after Student’s *t*-test) and L_P_2 14 days p.l. (14d, L_P_2 vs C_P_2 p = 0.001 after Student’s *t*-test). Different areas of interest of the pallial nerve analyzed in this study are indicated (see legend in **a**); see also legend of Fig. 1. See also text for detail and statistics.

We calculated the size of the scar by counting the number of cells both in the sham control nerve and at the same level in the lesioned nerve. At three days p.l. we found about 3000 cells per stack at the level of the lesion site (LL, see also Materials and Methods for identification of target areas); a number that appeared to be three times lower on the corresponding contralateral area (CL, 950 cells per stack; p = 0.043 after Student’s *t*-test; Fig. 5a). The number of cells forming the scar decreased to around 1800 cells per stack (LL) a week after injury (7 days p.l.) in the lesioned area, a number that appeared to be still different from the cells counted in control areas (CL: 1100 cells; p < 0.001 after Student’s *t*-test). Finally, a significant increase of the number of cells in respect to the contralateral area was observed in the peripheral side of the lesion (14 days p.l.; L_P_1 and L_P_2: 1900 and 2000 cells respectively; C_P_1 and C_P_2: 950 cells; L_P_1 vs C_P_1, p < 0.001; L_P_2 vs C_P_2, p = 0.019 after Student’s *t*-test; Fig. 5a).

The contribution of hemocytes to scar resulted not quantifiable under our experimental conditions. However, these appear to be the larger component. In fact, several cells identified by morphology as hemocytes contributed to scar formation at the lesion site (LL) and infiltrated between regenerating fibers of the central stump (Fig. 3b,c).

Numerous hemocytes also infiltrate the peripheral stump in contact with regenerating fibers at 14 days p.l.; these were not evident at earlier time-points in this area. Within the scar they appeared to undergo structural changes from the classical spherical form they retained in the vessels, to a spindle shape when they reached the site of lesion (LL, Fig. 3b). This observation is compatible with those reported by Féral^43^ during arm regeneration in the cuttlefish (*Sepia officinalis*) in which hemocytes (i.e. blood cells) migrated to the wound to form the blastema.

### Degeneration vs axonal regrowth

Regeneration occurred differently between the central and peripheral nerve stumps. The central stump was characterized by intensive axon growth already three days after surgery. On the other hand, the peripheral stump of the nerve towards the stellate ganglion showed mainly degenerative processes. The main axonal re-growth was observed in the proximal area of the central stump (Fig. 3a,b; areas corresponding to L_C_2 in Fig. 1) with fibers oriented in many different directions, revealing a disorganized pattern. The majority of the regenerating nervous fibers led toward the scar tissue, though some of them penetrated the scar.

The leading connective tissue still appeared not formed (Fig. 3b). The peripheral stump closest to the injury site was degenerating and we observed axons breaking in lumps, as originally described by Sereni^17^.

Seven days p.l. the outer connective tissue enveloping the nerve tightened around the cut stumps (Fig. 3e; corresponding to areas L_C_2 and L_P_2 close to the lesion in Fig. 1), apparently narrowing the nerve. Regenerating fibers at the level of the central stump continued growing in multiple directions, but most of them were observed to move past the cicatricial tissue, contacting the peripheral axons (Fig. 3f,g). Axon breakage in the peripheral stump involved a larger area than the previous stage (Fig. 3h). Multiple lumps formed by debris of degenerating axons appeared to be swollen. Neurofilaments (detected by NF200 antibody) appeared to accumulate towards these swollen neuronal terminal endings.

Significant changes in the nerve regeneration process were observed two weeks after the injury. At this time point, we observed a retraction of at least one of the two stumps, probably consistent with degeneration of the peripheral stump. This produced an apparent increased distance between the two stumps, compared to the first time-points investigated.

We observed numerous fibers directed toward the peripheral stump appearing well organized and forming a defined spike-like structure within nascent connective tissue (Fig. 3i). Nevertheless, the neural fibers in the central stump still generally showed a disorganized appearance. In the opposite stump, degeneration was still evident, although, many regenerating fibers protruded for several microns within the debris. These fibers appeared well-organized in bundles enveloped by connective tissue (Fig. 3j).

In all cases the two stumps were directed toward each other and made contact.

Regeneration/degeneration phenomena observed at the different time-points were also identified by considering the area occupied by the neuronal filaments. The major changes were observed in the lesioned nerve at the level of the central stump (L_C_2) seven days post lesion (L_C_2 vs C_C_2 neurofilament area, p < 0.001 after Student’s *t*-test; Fig. 5b) due to axonal regrowth in the central stump (Fig. 3a,b). Degeneration resulted in considerable axonal loss when compared with contralateral nerve (see Fig. 4), revealing a reduced neurofilament area (L_P_2 vs C_P_2, 3 and 7 days p.l., p < 0.010 after Student’s *t*-test; Fig. 5b).

Fourteen days after lesion a similar situation was found in the lesioned nerve at the level of the peripheral (L_P_2) stump (neurofilament area, lesioned vs contralateral side, p = 0.001 after Student’s *t*-test), but not when the central stump was considered (L_C_2 vs C_C_2, p = 0.924, NS after Student’s *t*-test). In the sham controls a constant number of fibers were detected at both sides (i.e. central vs periphery) and corresponding locations (Fig. 5b).

### Cell proliferation

**Figure 6:**
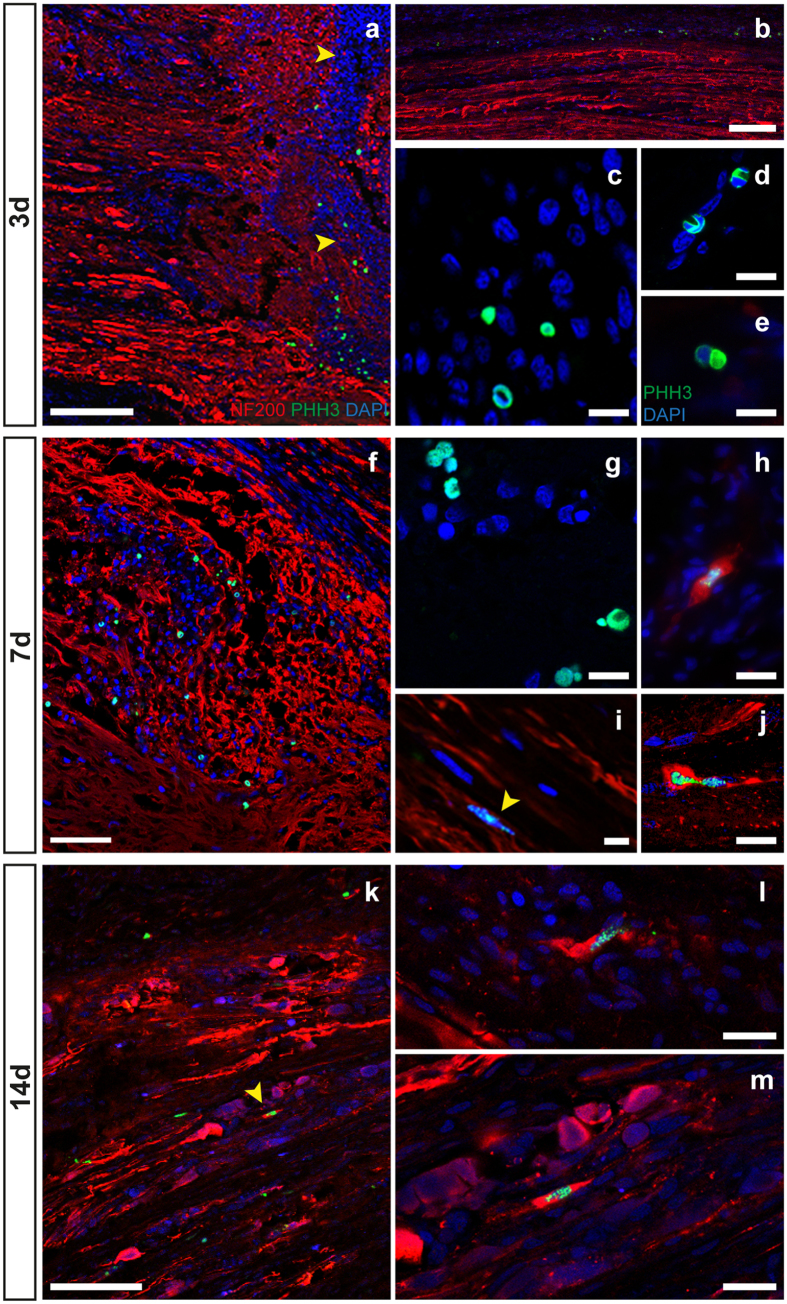
Cell proliferation. (**a–e**) In the scar tissue between the two cut stumps (marked with yellow arrows, **a**), many cells actively proliferate (green in **a–e**). (**b**) Proliferating hemocytes run inside the blood vessels which run along the pallial nerve. (**c–e**) Hemocytes are recognized by characteristic U-shape nuclei and the dimension of their nucleus. (**f,g**) Seven days post lesion hemocytes infiltrate among regenerating fibers of L_C_2 and proliferate. (**i**) Cells of the connective tissue, which envelop the nerve fibers, start proliferating inside the cut nerve. (**h,j**) Cells of the outer connective layer proliferate showing neurofilament marking. (**k–m**) 14 days post lesion, mitotic cells are found among degenerating nerve fibers, some of them showing neurofilament marking (marked with yellow arrow in **k**). Scale bars: (**a,b,k**) 100 μm; (**f**) 50 μm; (**c–e**; **g–j**) 10 μm; (**l,m**) 20 μm.

Large numbers of hemocytes were observed three days post injury contributing to the formation of the scar tissue between the two stumps. They also provided the main source of proliferating cells found at this time-point (Fig. 6a–e). We counted 20 PHH3 positive cells (from a total of 3000 per stack) at the lesioned site (LL, Fig. 5c; Number of PHH3 cells at LL vs CL: p = 0.008 after Student’s *t*-test). Mitotic hemocytes were also found within the blood vessel running into the central stump, leading to the injury and at the level of the connective tissue surrounding the nerve (Fig. 5b; number of PHH3 cells at L_c_2 vs C_C_2: p < 0.001 after Student’s *t*-test). The sham control showed some proliferating cells in the external connective layer, but no proliferating hemocytes were detected inside the nerve or in the inner blood vessels (Figs 4 and 5c).

Seven days after injury the number of proliferating cells remained similar (PHH3 *vs* total cells: 25, 1900 cells per stack, LL vs CL: p = 0.001 after Student’s *t*-test) to the number observed during the previous time-point. PHH3 positive cells did not appear restricted to the lesioned site, but rather were expanding towards new growing fibers (L_C_2, PHH3 vs total cells per stack: 46, 1500 respectively; L_c_2 vs C_C_2, p = 0.001 after Student’s *t*-test; Figs 5c and 6f). At the same time-point we also observed other proliferating cells with morphological features typical of the connective tissue. These were characterized by larger elongated nuclei and were positioned within the tissue around nerve fibers (Fig. 6i).

Similar cells occasionally appeared in the sham control nerves and in uninjured nerve (data not shown), suggesting a basal level of proliferation of the connective tissue. However, these numbers were low compared to the injured nerve. We found on average zero to three proliferating cells per stack in the sham nerve (Fig. 4), and between zero and one in the uninjured nerve.

A large number of proliferating cells were found inside the lesion site 14 days after injury (PHH3 *vs* total cells: 25, 1800 cells per stack; LL vs CL p < 0.001 after Student’s *t*-test). A similar number was also detected in the peripheral stump (PHH3 positive *vs* total cells per stack, L_P_2: 28, 2000; L_P_1: 6, 1900; p = 0.009 after Student’s *t*-test; Figs 5c, and 6k). The great majority of these cells were identified as connective tissue-like cells and hemocytes.

Finally, we detected proliferating cells at the level of the peripheral stump. These were also positively marked with the neuronal marker NF200. These cells appeared to be scattered between both degenerating and regenerating fibers (Fig. 6l,m).

Some mitotic cells appearing within the external connective layer were also positive for NF200. The latter were identified at all the time-points in both the injured nerve (Fig. 6h,j) and the sham control.

In any case, no NF200-positive cells were ever found inside the control nerve.

## Discussion

Our findings extend those previously reported to occur after *O. vulgaris* pallial nerve lesion and subsequent regeneration (Table 1). After complete transection of the pallial nerve hemorrhagic areas appearing on the lesioned side were immediately followed by cicatricial tissue formation. A scar is formed between the two stumps, but it does not represent an inhibitory environment to the nervous fibers that start to regenerate from the central stump and cross the injury site to reach the peripheral side. Although the nature of scar cells remains unknown, we were able to recognize hemocytes contributing to scar tissue. They were identified by their characteristic round shape and U-shaped nuclei (Fig. 6d), and appeared to originate by the damaged vessels during surgery.

The intense vascularization in the vicinity of pallial nerves^42^ supports this view. The stellate ganglia are supplied by right and left pallial arteries that branch from the anterior aorta and that run dorsally to two pallial veins. In addition, a blood vessel runs within the pallial nerve itself, and likely contributed to hemocytes release in the area of injury.

Hemocytes are the only cell type reported in the circulating hemolymph of *O. vulgaris* and they possess phagocytic activity^44^,^45^, thus supporting the view that their presence in areas closed to the lesion, and between degenerating and regenerating neuronal fibers, may in part be related to active debris removal, as originally observed by Sereni^17^.

**Figure 7:**
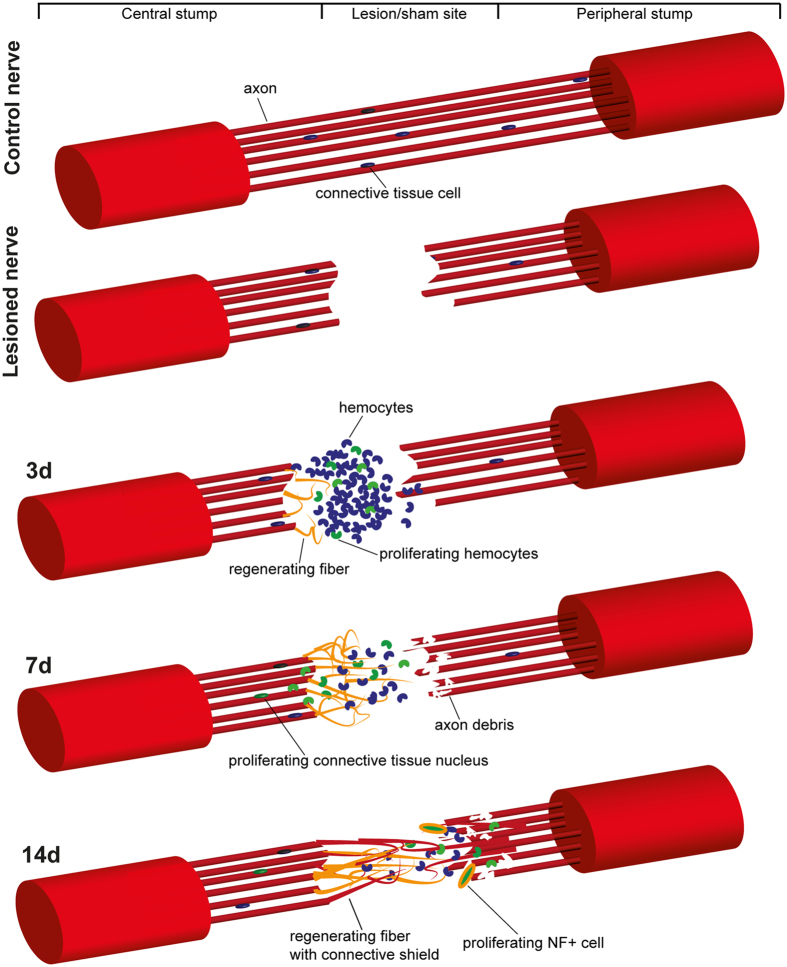
Graphical overview of the processes occurring following octopus pallial nerve lesion. Hemocytes are involved in scar formation between nerve stumps; they actively proliferate in LL three days post injury. Regenerating fibers are found in the central stump, growing in several directions. Seven days after lesion fibers are able to grow across the scar toward the peripheral stump. Many connective tissue cells proliferate inside the nerve. A spike-like structure forms two weeks post injury in the central stump, due to connective tissue guide. In the peripheral stump, so far showing mainly axon degeneration, bundles of regenerating axons appear, enveloped by new forming connective tissue. See text for details.

As schematized in Fig. 7, hemocytes have a leading role in the regenerative process of the pallial nerve. They first migrate to the lesion site and spread among scar and regenerating fibers of the central stump. Mitotic hemocytes are recruited to the lesion through systemic blood circulation, travelling into the blood vessel inside the central stump. No mitotic hemocytes were found in the contralateral nerve, thus suggesting that some chemo-attractive signals are released in the site of lesion and are responsible for attracting proliferating hemocytes as described by Féral^43^. Interestingly, these signals apparently cannot be triggered by a generic insult (e.g. muscles or connective tissue lesion), but require injury of the nerve or of the blood vessel running inside the nerve, as proven by the fact that in sham controls, where the nerve was approached, but left intact, mitotic hemocytes were not found. Also consistent with a regenerative role for hemocytes is the fact that proliferating blood cells are initially found mainly in the central stump, where a substantial tangle of new fibers is growing. In the opposite stump little sign of regeneration is visible and the number of proliferating hemocytes is more restricted. It is possible that hemocytes may release factors that foster regeneration of axons; this assumption seems to be further supported by the fact that at 14 days after lesion numerous mitotic hemocytes are found among regenerating fibers of the peripheral stump.

In parallel, mitotic cells belonging to connective tissue also appear soon after nerve lesion. Their number also increases with time, following a similar pattern of compartmentalization of hemocytes: first in the central stump and only later in the periphery.

In the last time-point observed (14 days post lesion), mitotic cells positive for neurofilament marker were found among the degenerating fibers and regenerating bundles of the peripheral stump (Fig. 6l,m). Although their nature and function is still unknown, the expression of neurofilament marker in these cells might indicate a process of differentiation of unlabeled stem/progenitor cells (or glial cells) present at the early stages of pallial nerve regeneration. Connective tissue cells of the external layer also proliferate and express NF200 marker, thus supporting the view that glial cells might be involved in the process.

In the sham control, the nerves showed no morphological alterations and just a few mitotic cells; these were primarily in the outer connective tissue.

There is no evidence of available markers of cephalopod’s glial cells. We tested a series of antibodies (i.e. vimentin, glial fibrillary acidic protein; data not shown) without being able to confirm previous data^46^. Furthermore, there is no evidence of the presence of ortholog transcripts for glial fibrillary acidic protein in *O. bimaculoides* genome^47^ or in *O. vulgaris* transcriptome^48^.

Our observation of connective tissue cells behavior, after nerve damage, and the finding of myelin-related proteins found to be involved in regeneration in other invertebrates^49^,^50^,^51^, support our hypothesis of connective cells may act as stem/progenitor cells (or glial cells), even though this need to be further investigated and might be the subject of future studies.

Axon outgrowth in the central stump after complete transection is quick and, at least initially, very disorganized. Old outer connective tissue tightens around the cut nerve ends while inner connective sheaths do not grow together with regenerating fibers. Only later, organized connective sheaths wrap fibers, forming a spike-like structure in the central stump, directed toward the peripheral stump. Fibers of the peripheral stump, instead, degenerate and form debris, which gradually extend in the nerve from the site of lesion toward the stellate ganglion. A few days later, the peripheral this stump shows evident signs of regeneration, with new fibers growing into fascicles and tightly enwrapped in connective tissue (Fig. 7). Detection of NF200-positive proliferating cells at the level of the peripheral stump and between degenerating and regenerating fibers suggests that neuronal stem/progenitor cells also contribute to nerve regeneration.

Thus, nerve regeneration in the octopus involves at least *i.* axonal re-growth and *ii.* neuronal stem/progenitor cell proliferation and differentiation.

As summarized in Table 1, we observed partial functional recovery between seven and 14 days post lesion in contrast to what reported by previous studies^14^,^17^. This recovery pertains only to skin pattern and not breathing due to impaired mantle contraction. Indeed, while the animals showed again some ability in modulating chromatophore contraction/relaxation, mantle muscles contraction remained inhibited at the level of the lesioned side. This might be explained by a local control of chromatophores, exerted by light on skin receptors rather than re-innervation of target tissues, as already suggested by Packard and Brancato^52^.

Unlike the response of mammalian spinal cords to injury, where a glial scar forms and structural reconnectivity is inhibited^53^, several aspects of the observed response in octopuses mirror the regenerative response of mammalian peripheral nerves, which, following crush injury or acute transection and repair, is often successful.

The response of peripheral nerves to injury has been well-characterized^54^,^55^,as reviewed in Hall^56^. Briefly, several structural and biological changes occur in the severed nerve stumps within 1–3 days of injury, including cytoskeletal destabilization and organelle accumulation in the proximal stump, and Wallerian degeneration in the distal stump. The primary structural changes to the distal stump are the loss of nerve fibers and dedifferentiation and compaction of Schwann cells and basal lamina into Bands of Büngner^57^,^58^,^59^,^60^,^61^. Also present in these bands are various proteoglycans and an organized (non-fibrotic) collagenous matrix, which collectively provide well-organized tracks for regenerating axons. Proximal stump outgrowth, axonal sprouting, and extension begin within 3–5 days following injury, and axons that extend into the distal stump within 1–2 months thus experience a highly favorable regenerative environment, which is further enhanced by increased Schwann cell expression of chemoattractive growth factors and their receptors^62^,^63^. While Bands of Büngner are not readily apparent in the regenerating octopus pallial nerve, the progressive increase in structural organization in the early days following injury suggests the creation of a favorable structural environment for axonal regrowth. Importantly, in both models, as proximal axons enter the distal stump, normal structure is restored in the vicinity of regenerating axons.

In parallel to structural changes, degenerating mammalian nerves also experience the infiltration of a variety of cells at the injury site. Indeed, bone-marrow macrophages reach the site of lesion in a few hours, and within one to two days, macrophages are recruited from systemic circulation, attracted by inflammatory cytokines and chemokines released by resident macrophages^54^. Among other functions, these macrophages are responsible for sequestering and eliminating or recycling myelin debris, which appear to be the role of hemocytes in *O. vulgaris*.

Compellingly, recent work in mammals suggests an important role for stem/progenitor cells, which may be recruited from surrounding muscle or the vasculature, in improving nerve regeneration^64^. Proliferation and conversion of these cells into nerve cells such as Schwann cells appears to be an essential aspect of the regenerative response. Our working hypothesis is that these may represent conceptual analogy with proliferating connective tissue cells we observed in the octopus.

Cumulatively, then, successful repair in both octopus and mammals appears to be guided by effective innate-immune response and the timely intervention of Schwann cells, fibroblasts, endothelial cells, and the molecules they produce^54^,^65^,^66^. This has been also suggested in cephalopods by Féral^43^,^67^,^68^.

Although further investigation is required to better characterize the regenerative process in octopuses, these results represent an important starting point to understand the mechanisms involved in nerve regeneration in *Octopus*. Hemocytes and connective tissue cells contribution was described here to greater extent than original observations by Sereni and Young^17^, underlining the involvement of immune cells and glia for *O. vulgaris* as occurs in all vertebrate models of nerve regeneration. Two weeks, indeed, appear sufficient to organize and build the directional and leading scaffolding in both stumps, allowing them to grow toward each other, overcome the scar and, in some cases, to obtain a partial recovery of the chromatic function. We cannot exclude that the functional recovery of the body patterning in the octopus maybe also facilitated by phenomena (e.g. local network control) not necessarily related to structural regeneration, as suggested by Packard and Brancato^52^.

## Materials and Methods

### Animals

Adult *Octopus vulgaris*, both sexes (body weight: 250–350 g; N = 12, ♂ = 5 ♀ = 7) caught from the Bay of Naples were kept under standardized conditions^34^,^69^ in the laboratory. Experiments were conducted during spring of the 2013 (sea water temperature range: 18–20 °C). All the animals were fed with crabs once a day. Octopuses for this study were selected for the absence of any regenerating sign or any kind of lesion, and appearing exploratory driven and healthy^32^,^33^. Experiments with live octopuses were carried out before transposition of Directive 2010/63/EU in Italy. Although no authorization was required, all procedures were performed in order to minimize the pain and distress of the animals involved^29^,^30^,^31^,^70^.

### Nerve transection

*O. vulgaris* were anesthetized by immersion in 3.5% MgCl_2_ in sea water for 15 minutes, which produced complete relaxation and immobility of the animals^71^. Anesthetized animals were placed on a surgery table, positioned on a dissecting tray containing the anaesthetic solution. Octopuses were turned on their ventral side, the mantle slightly overturned to expose the nerve and the stellate ganglion nearby; a scalpel was used to make an incision on the internal side of the mantle cutting the skin, muscle and connective tissue enwrapping the pallial nerves. In this way both nerves (left and right side of the animal) were exposed using a skin hooklet. To standardize the site of the injury along the nerve, the diameter of stellate ganglion was measured for each animal, and the same length computed as distance along the nerve to set the site of severing (identified as LL in our experiments; see also Fig. 1). Left-side nerve remained intact and gently positioned back on its natural position thus serving as a sham control. The right-side pallial nerve was hold with the hook and completely transected using fine scissors. The entire operation lasted less than five minutes.

The completeness of the transection was verified by visual inspection under a stereo microscope. Uninjured samples belonging to other three animals were also collected to assess the effect of lesion on tissues surrounding the nerve.

Following surgery the animals were returned to their tanks and allowed to recover^71^,^72^. Full recovery was based observing re-acquisition of the normal posture, regular breathing rate and return to den; this usually requires less than 30 minutes, and a full predatory response was recorded 60 minutes later^30^,^73^.

### Behavioral observations and animals care

After recovery, octopuses were maintained in experimental tanks and fed on live crab (*Carcinus maenas*) every day following procedures described in Amodio *et al*.^34^. Predatory responses were videorecorded by remote controlled digital video cameras (Panasonic HDC-SD80), which were hidden from each animal’s view. Each presentation lasted a maximum of two minutes (ceiling latency to attack: 121 s) and a failure to attack within this period was classified as “no attack”^32^,^34^,^69^. Behavioral observations were carried out at the same time of the day, in the afternoon. Video recordings of operated animals were examined to analyze behavior and to detect changes in octopus appearance between the lesioned and the control sides. In particular, chromatic and textural patterns were noted daily during the attack behavior and at rest, for at least 10 minutes before and after the prey presentation in the octopus tank.

From video-recordings, two independent observers deduced ***i.*** the latency to attack the prey; ***ii.*** the body pattering and the approximate areas of blanching of the skin (mantle and any other body part) according to the descriptions provided by other authors^14^,^21^,^22^. Body patterning observed during the analysis of video-recordings was coded following Borrelli *et al*.^3^. Behavioral observations also served as assessment of health and welfare of animals according to principles stated in Directive 2010/63/EU; Signs based on appearance, behavior and physiology were searched from a checklist as part of health monitoring program and eventually recorded^31^.

### Collection of samples and humane-killing of octopuses

Animals were humanely killed at three different time-points: three, seven and 14 days after injury. At the selected time-points the octopuses were deeply anesthesized (>30 min) by immersion in a 3.5% solution of magnesium chloride hexahydrate in seawater; death was confirmed by transection of dorsal aorta^31^,^71^.

On the surgery pad, a clamp was used to pinch and hold the pallial nerve during harvesting. Both pallial nerves (cut and sham or control) were collected together with the stellate ganglion and surrounding tissues.

### Frozen and paraffin sections

Samples were fixed in paraformaldehyde (4% PFA in sea water; for 1 h 30 min), washed in PBS (pH 7.4) and rinsed over night at 4°C. They were cryoprotected in sucrose (30% in PBS; pH 7.4) until tissue sinking, and frozen using tissue freezing and blocking medium (OCT; Leica Biosystems). Longitudinal 30 μm thick slices were obtained using a cryostat (Leica CM3050 S). For paraffin sections, samples were dehydrated through an ascending series of alcohol, cleared in xylene, immersed in paraffin and then cut into 5 μm thick sections.

### Immunohistochemistry and histology

Cryostat sections were used for immunohistochemistry. They were air dried for 1 h, washed in PBS and Normal Goat Serum (NGS) 5% in PBT (PBS + Tween 0.1%) was used as a blocking agent. Slices were then incubated with primary antibodies, diluted in NGS 1% in PBT overnight at 4°C. Anti-Neurofilament 200 Sigma N5389 (NF-200, 1:2000), and anti-Phospho-Histone H3 Sigma H9908 (PHH3; 1:600) to label axons and detect cell proliferation, respectively were utilized. Sections were then washed with PBT and then incubated with secondary antibodies (1:250; respectively Alexa Fluor goat anti-mouse IgG1 594, and Alexa Fluor goat anti-rat IgG (H + L) 488) for 1 h at room temperature. The omission of each primary antibody was used as negative control for each immunostaining. Following washing in PBT, DAPI (diluted 1:1000 in PBS) was used to counterstain nuclei. Paraffin sections were stained for Masson’s trichrome^74^ after paraffin removal and rehydration in an ethanol series.

### Confocal imaging

Sections labeled with fluorescent dyes were imaged on a Zeiss LSM 710 laser scanning confocal microscope using a 20 × air objective. Z-stacks of five areas of the damaged nerve were taken.

The analysis of the events occurring after surgery focused on five areas for each pallial nerve (Fig. 1): the site of lesion (LL), a distal and a proximal area in the central stump (respectively identified as L_C_1 and L_C_2), and in the peripheral stump (L_P_1 and L_P_2) of the transected nerve. Analogous areas were identified in the control nerve preparation: corresponding lesion site (CS), a distal and a proximal area in the central ‘stump’ (C_C_1, C_C_2), and at the level of the peripheral ‘stump’ (C_P_1, and C_P_2).

### Morphology and anatomical description

We follow morphological and event descriptions as provided by Young and coworkers^15^,^17^,^20^,^24^. The two stumps have been identified as belonging to central and peripheral sides of the pallial nerve, respectively. According to modern literature on nerve regeneration, these should be named respectively proximal and distal, but here we refer to the classic terminology.

### Image and Data analysis

Images were processed with IMARIS 64 7.5 (Bitplane Inc.) for nuclear and mitotic cells counting and neurofilament area calculation. The analysis has been carried out from ten serial sections (stacks, 20 μm thick) of each area of interest (N = 9, three for each time-point). Statistics and box-and-whisker plots were generated using SPSS version 14.0 (SPSS Inc). Data were tested for normality and Student’s t-test was applied according to Zar^75^. Differences were considered significant at p < 0.05.

## Additional Information

**How to cite this article**: Imperadore, P. *et al*. Nerve degeneration and regeneration in the cephalopod mollusc Octopus vulgaris: the case of the pallial nerve. *Sci. Rep.* **7**, 46564; doi: 10.1038/srep46564 (2017).

**Publisher's note:** Springer Nature remains neutral with regard to jurisdictional claims in published maps and institutional affiliations.

## Figures and Tables

**Table 1:** An historical overview of phenomena described to occur after lesion of the pallial nerve in *Octopus vulgaris*.

Structure and/or Events		Young, 1929^19^	Sereni & Young, 1932^17^	Young, 1972^20^	Sanders &Young, 1974^14^	*This study*
Nerve Fibers	Degeneration	**√**	**√**	**√**	**√**	**√**
	Regeneration		**√**	**√**	**√**	**√**
Scar formation			**√**			**√**
Hemocytes	Infiltration among nerve fibers		**√**			**√**
	Proliferation		**√**			**√**
	Phagocytosis		**√**			
	Contribution to scar		**√**			**√**
Connective tissue cells	Proliferation					**√**
	Neuronal marker expression					**√**
Neuronal Cells	Presence in the nerve					**√**
	Proliferation					**√**
First signs of functional recovery of body patterning					**30d**	**7d**

The information obtained in this study is summarized here as comparison. Young^19^ observed only degeneration of both stumps within the first 10 days after lesion (d), no further degenerative phenomena were reported till the end of the experiments (40 days in Young^19^). Sereni and Young^17^ observed “amoebocytes” infiltration into the cut stumps, especially the peripheral one, where they appear to phagocytose actively. Young^20^ reported a “vigorous” regeneration originating from the peripheral stump of the pallial nerve, and suggested this due to the afferent fibers from the periphery.

Some of the most significant events occurring after lesion of the octopus pallial nerve are described for the first time in this study.
